# Improvement of a Switchable Wide-Incident-Angle Perfect Absorber Incorporating Sb_2_S_3_

**DOI:** 10.3390/ma18235305

**Published:** 2025-11-25

**Authors:** Yaolan Tian, Guoxu Zhang, Yan Li, Mei Shen, Yufeng Xiong, Ting Li, Yunzheng Wang, Xian Zhao, Changbao Ma

**Affiliations:** 1 Center for Optics Research and Engineering, Shandong Provincial Key Laboratory of Laser Technology and Application, Key Laboratory of Laser & Infrared System, Ministry of Education, Shandong University, Qingdao 266237, China202334052@mail.sdu.edu.cn (G.Z.); xiongyufeng_1995@126.com (Y.X.);; 2 College of Integrated Circuits, Shenzhen Polytechnic University, Shenzhen 518052, China; 3 Suzhou Research Institute of Shandong University, Room1107, Building B of NUSP, NO. 388 Ruoshui Road, SIP, Suzhou 215123, China; 4 Shenzhen Research Institute of Shandong University, A301 Virtual University Park in South District of Shenzhen, Shenzhen 518055, China

**Keywords:** perfect absorption, phase change materials, Sb_2_S_3_, metasurfaces

## Abstract

Active metasurfaces, whose optical properties can be tuned by an external stimulus such as electric or laser pulses, have attracted great research interest recently. The phase change material (PCM), antimony sulfide (Sb_2_S_3_), has been reported to modulate resonance wavelengths from the visible to the infrared. Here, we present a purely numerical study of an active and nonvolatile narrow-band perfect absorber in the infrared region based on a nanostructured metal–insulator–metal (MIM) metasurface incorporating Sb_2_S_3_. The proposed absorber exhibits a high quality factor and achieves near-unity absorption at resonance wavelengths. In addition, the absorption spectrum can be dynamically modulated by the phase transition of Sb_2_S_3_, with a modulation range approaching 1 μm. Moreover, the designed absorber shows insensitivity to the angle of incidence. This study offers a feasible strategy for developing Sb_2_S_3_-integrated metasurface perfect absorbers with potential applications in selective thermal emitters and bolometers.

## 1. Introduction

Metasurfaces, consisting of ultrathin artificial arrays of sub-wavelength structures, have attracted a lot of attention due to their special electromagnetic responses and customizable effective physical properties. The artificial structures can interact with incident light, modify the properties of the scattered electromagnetic waves, and enable precise manipulation of the optical field at a sub-wavelength scale [1,2]. Applying the unique electromagnetic functions of metasurfaces, exceptional optical phenomena such as negative refraction, polarization conversion, and perfect absorption have been realized [3,4]. Consequently, a tremendously revolutionary change has been brought to traditional micro-nano photonic devices, developing lots of new features and devices such as meta-lens [5], beam steering [6], optical imaging [7], and perfect absorber (PA) [8,9]. Among them, PA can achieve near-unity absorption and enhance the capturing efficiency of incident energy. Hence, they have been rapidly developed in recent years due to their important applications in photonics and optoelectronics fields.

PAs could be generally classified into narrow-band, multiband, and broadband types, based on the number and bandwidth of absorption peaks. The first type can be directly used as optical switches or bolometric devices [10], and the last type can be applied in bolometric devices and solar thermal applications [3,11]. Various configurations of narrow-band PAs have been demonstrated, such as gold lattices including thick GST films [12], nanodisk-based metasurfaces [13], dielectric cylinder arrays [14], and metallic square arrays [15]. Similarly, broadband PAs have been realized using a metal–dielectric–metal (MIM) structure [3] and in the dielectric-nanodisk-based metasurface [16], operating across a wide spectral range from visible to infrared wavelengths. Generally, the absorption spectrum of PAs can be easily regulated by adjusting the geometric parameters and the applied materials [17]. However, once devices were fabricated, the geometry and the materials used became unchangeable; consequently, the absorption spectrum is non-switchable at a certain incident angle. To enable dynamic spectral tuning, actively tunable materials such as graphene [18], VO_2_ [19] and conducting oxides [20] have been explored, allowing electrical modulation of absorption properties. Additionally, ultrafast modulation of the spectral response, phase, and polarization of light could be achieved by hot-electron assistance [21,22]. However, these methods require sustained stimulation, which is not environmentally friendly.

Recently, phase change materials (PCMs) such as Ge_2_Sb_2_Te_5_ (GST) [23,24,25], Ge_2_Sb_2_Se_4_Te_1_ (GSST) [26,27] and GeTe [28] have been integrated into metasurfaces to actively modulate the absorption spectrum. They exhibit many intriguing advantages, such as nonvolatile, rapid, and reversible switching between the amorphous and crystalline states by electric or optical pulses, and a large tuning range of optical properties [29,30]. For example, GST provides a strong refractive index contrast (Δn ≈ 2) and a mature integration process, and GSST offers substantial thermal robustness due to a large crystallization temperature [31]. However, GST has a large extinction coefficient in the visible and infrared ranges, especially in the crystalline state, which could highly reduce the absorption peak of GST-integrated PAs due to the energy loss induced by its electric permittivity [13]. Recently, Sb_2_S_3_ has attracted growing research attention, not only for its nonvolatility, but also for its low optical losses in the infrared region. Currently, Sb_2_S_3_ is promising for incorporating into reconfigurable on-chip nanophotonic devices and programmable optical devices [32,33].

In this work, we propose a Sb_2_S_3_-integrated metasurface PA as a narrow-band perfect absorber. The absorber is composed of a square metal nanodisk array on top of a continuous metal film separated by a thin Sb_2_S_3_ layer, i.e., a MIM structure made of either Al or Au as passive metal layers. We numerically study the fundamental optical response of our designed PAs in the infrared region, and they perform perfect narrow-band absorption, with peak absorbance exceeding 94% under two phases of Sb_2_S_3_. Furthermore, we investigate the physical mechanism of the perfect absorption and the effects of the geometric parameters and incident angle on the absorption spectrum.

## 2. Simulation Model

**Figure 1:**
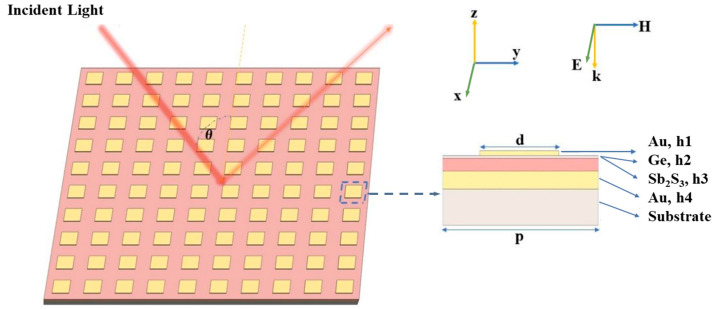
Schematic illustration of the designed PA. Left: The proposed PA based on Sb_2_S_3_ metasurface with incident and reflection light. Right: A unit cell of the PA with the definition of layer thickness and electromagnetic field direction. h1, h2, h3, and h4 stand for the thicknesses of the top Al/Au layer, the Ge layer, the Sb_2_S_3_ layer, and the bottom Al/Au layer. p stands for the periods in the x and y directions, and d stands for the side length of the top metal.

The proposed MIM structure of PA is schematically depicted in Figure 1. The basic design for each unit is composed of three layers on a glass substrate: a h4-thick metal layer as a mirror to prevent transmission, a layer of h3-thick Sb_2_S_3_, and a h1-thick square metal layer with side length of d. Additionally, Sb_2_S_3_ thin film is covered by a protective layer of Ge (h2-thick) to avoid sulfur loss during switching. The unit lattice periods along both the x- and y-axes are equal to p. Generally, Au is employed for such MIM configurations due to its stability in many complex environments. On the other hand, it is also common to employ Al in the industry, a good choice for balancing affordability and performance. Therefore, we simulate metasurface absorbers incorporating either Au or Al as the metallic components.

Here, h1 = 40 nm, h2 = 15 nm, h3 = 70 nm, h4 = 100 nm, and d = 0.8 μm. For Al-incorporated PA, p is set to be 1.5 μm, while p = 1 μm is selected for Au-integrated PA, due to the different electric properties of Al and Au. In this paper, the FDTD method is mainly employed to simulate and optimize the metasurface absorber. The dielectric function of Al is simulated on the basis of the CRC model [34], while that of Au is obtained from Palik’s handbook [35]. The refractive index and extinction coefficient of Sb_2_S_3_ are from Adam’s paper [32]. The refractive index of the Ge layer is also obtained from Palik’s handbook [35]. All the dielectric data were fitted by a sixth-order polynomial to achieve low fitting errors. Perfectly matched layers are applied along the z direction, and periodic boundary conditions are set to the side surfaces of one metasurface unit in the x and y directions, respectively. The computational domain size is p × p × 2.5 μm^3^, including all PA layers, top air layer, and bottom substrate layer. The domain size in the z direction was proven to be sufficient by choosing different lengths. The grid step in the Ge layer is chosen to be 5 nm along the z direction and 10 nm along the x and y directions, corresponding to about 400 points per wavelength. PA is illuminated by a normally incident plane wave with unitary electric field amplitude and linear polarization along the x direction and wave vector along the z axis. In this work, we defined the x-polarization as TE polarization and the y-polarization as TM polarization to conveniently describe the direction of the electric field. The reflection spectrum (R) was directly monitored by a power monitor in our simulations, while the absorption (A) can be deduced by A = 1 − R, since the transmission (T) is zero, owing to the opaque 100 nm-thick metal mirror.

## 3. Results and Discussion

### 3.1. Narrow-Band Infrared Absorption

**Figure 2:**
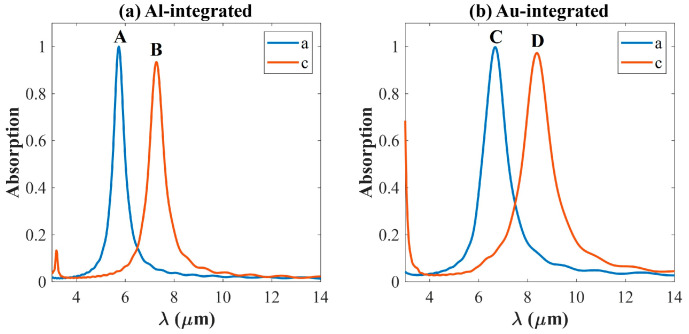
The absorption spectra of Al-integrated PA (**a**) and Au-integrated PA (**b**) under both amorphous and crystalline Sb_2_S_3_ states. A, B, C and D indicate the absorption peaks within 3–14 μm.

Figure 2 demonstrates the simulated absorption spectrum for both aSb_2_S_3_ (blue line) state and cSb_2_S_3_ (red line) states at a normal incident TE plane wave as a function of wavelength. It clearly demonstrates narrow-band perfect absorption, whose absorption peak is red-shifted with a slight peak change when Sb_2_S_3_ transfers from an amorphous state to a crystalline state. In Figure 2a, the absorption spectrum of the Al-based absorber in the amorphous state displays a resonant peak at 5.73 μm with a maximum absorption of 99.9%. Its quality factor is as large as 11.3, larger or comparable to the reported values in Refs. [12,15,28,36]. After crystallization, the absorption at 5.73 μm decreases significantly to 4.58%, and a new resonance emerges at 7.28 μm with a peak absorption of 93.5%, compared to the original 5.31% at this wavelength in the amorphous state. Figure 2b illustrates the absorption spectra of Au-integrated PA. Obviously, the resonance peaks are broadened and red-shifted compared with those shown in Figure 2a. This result is similar to what has been shown by the Mie scattering theory that the optical resonance of a dielectric nanosphere also red-shifts with the increasing refractive index of the material [37]. For Au-integrated PA, the resonance peak under aSb_2_S_3_ state is as high as 99.8%, located at 6.68 μm, which reduces to 12.6% as Sb_2_S_3_ crystallizes. Meanwhile, the absorption at 8.38 μm jumps from 12.6% to 97.3%.

The peak absorptions in Figure 2 are higher than 93%, larger than or comparable with the reported absorptions in Refs. [12,15,28]. The top metal nanodisk array serves as an optical antenna, interacting with the incident light and exhibiting resonance at specific wavelengths [38]. Simultaneously, the incident light is multi-reflected inside the MIM cavity, and can achieve a resonance at certain frequencies [39,40]. Therefore, the absorption peaks are attained as a coupling effect between the top antenna resonance and the cavity resonance [13,16]. As the incident frequencies approach the resonance frequencies, the amplitude of the electric field is maximized, while as the incident frequencies deviate from the resonance frequencies, the amplitude is apparently reduced [41,42].

The modulation caused by the phase transition of Sb_2_S_3_ proves that the Sb_2_S_3_ layer embedded between the two metal layers plays an important role in controlling the resonance conditions for perfect absorption. This behavior can be attributed to the substantial change in the dielectric constant of Sb_2_S_3_ during the phase transition, which strongly perturbs the resonant mode. The wavelength shift between two resonance peaks under two different states of the same PA structure almost reaches 1.5 μm. And the huge change in the absorption between the amorphous state and crystalline states makes it possible to be applied as a high-contrast optical switch. Furthermore, the profile of two lines under crystalline state rising up at 3 μm indicates that more peaks exist in a wavelength range smaller than 3 μm, which is also proven in Section 3.3 two peaks show up as d is larger than 1.2 μm. Detailed analysis of these resonant modes and their corresponding electric and magnetic field distributions is provided in the Supporting Information.

### 3.2. Physical Mechanism of Narrow-Band Infrared Absorption

**Figure 3:**
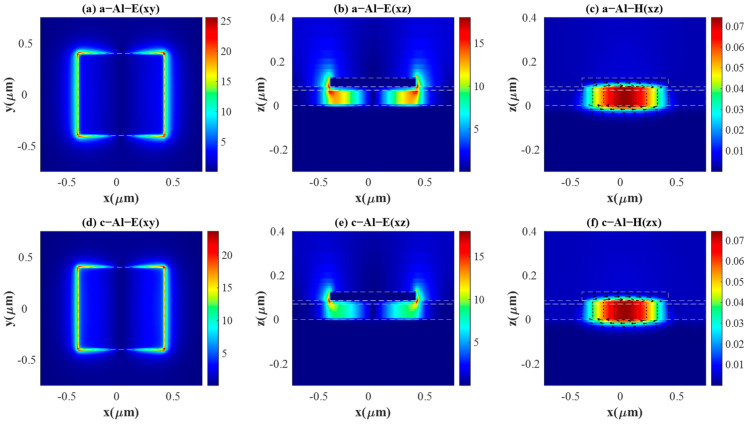
The electric field and magnetic field distributions under a TE incidence for Al-integrated PA. (**a**–**c**) show E(xy), E(xz), and H(xz) with the wavelength of 5.73 μm under aSb_2_S_3_ state. (**d**–**f**) show E(xy), E(xz), and H(xz) with incident wavelength 7.28 μm under cSb_2_S_3_ state. The arrows in (**c**,**f**) stand for displacement current.

**Figure 4:**
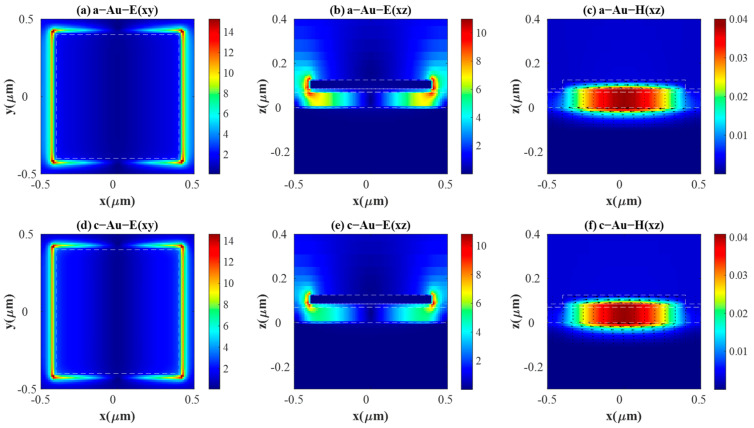
The electric and magnetic field distributions under a TE incidence for Au-integrated PA. (**a**–**c**) show E(xy), E(xz), H(xz) with the incident wavelength of 6.68 μm under aSb_2_S_3_ state. (**d**–**f**) show E(xy), E(xz), H(xz) with incident wavelength 8.38 μm under cSb_2_S_3_ state.

To gain further insight into the physical mechanism underlying the perfect absorption of the purposed PAs, we investigate the near-field electric field distribution E(xy) in the x-y plane on the top surface of layer Ge and E(xz) in the x-z plane at the center section (y = 0), the magnetic field distribution H(xz) in the x-z plane at y = 0 and the displacement current of the resonance peaks (A, B, C, D) in Figure 2. Figure 3 demonstrates those distributions corresponding to peak A and peak B, while Figure 4 presents those distributions corresponding to peak C and peak D. The color represents the magnitude of the electromagnetic field, and the arrow indicates the displacement current, with its length proportional to the magnitude. In Figure 3 and Figure 4, (a–c) present E(xy), E(xz), and H(xz) under aSb_2_S_3_ state; (d–f) show E(xy), E(xz), and H(xz) under cSb_2_S_3_ state. It is obvious that the distributions of the electromagnetic field are similar for aSb_2_S_3_ and cSb_2_S_3_ due to the fact that they all correspond to the results at the resonance peaks.

Figure 3a,b,d,e and Figure 4a,b,d,e exhibit a dipolar electric field pattern, consistent with the excitation of an electric dipole resonance [16,43]. When the light illuminates the PA, localized surface plasmon resonances (LSPRs) are excited between the two metal layers [41,44]. It is clear that E(xy) is mainly confined within the Sb_2_S_3_ layer and the Ge layer, as well as around the four corners of the top antenna. Thus, the energy carried by the electric field is well confined. The intensity of the electric field along x = 0 is zero, which can be called a node. The presence of a single node in the magnetic field H(xz) distribution is characteristic of the fundamental (first-order) resonant mode, shown in Figure 3c,f and Figure 4c,f. H(xz) is also a magnetic dipole, caused by the circular displacement current, and the concomitant coupling exists between surface plasmons counter-propagating on the two adjacent antennas [13,42]. Accordingly, energy carried by the magnetic field is completely confined within the interior of the cavity formed by metal–Sb_2_S_3_–metal. In addition, it can minimize reflectance and generate a highly efficient thermal emissivity [45]. In conclusion, the absorption peak is initiated by the electric dipole and the magnetic dipole.

### 3.3. The Influence of Geometric Parameters

We investigate the effects of different geometrical parameters on the absorption of the proposed PA, since the resonant wavelength of the MIM structure can be effectively influenced by size, shape, and thickness [16]. In this section, we focus on the effects of Sb_2_S_3_ thickness (h3) and the side length (d) of the top metal antenna under the aSb_2_S_3_ state and cSb_2_S_3_ state for both Al- and Au-integrated PAs. Only one parameter is changed at each simulation.

**Figure 5:**
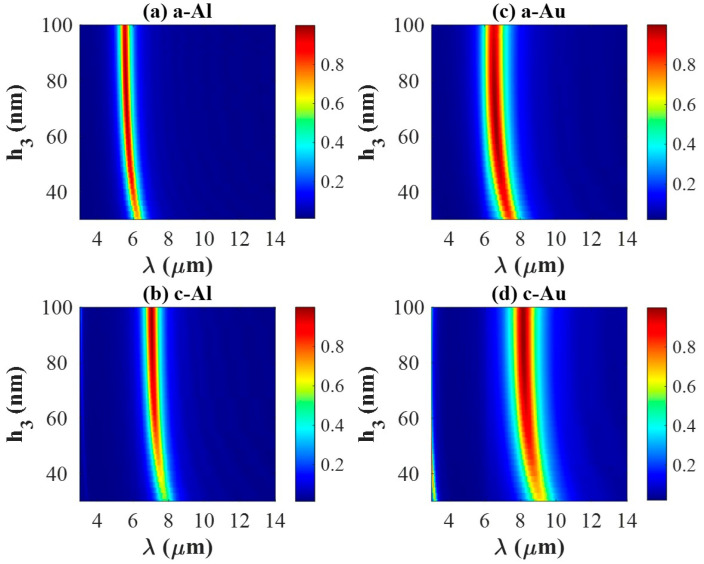
Absorption spectra for Al-integrated PA in (**a**,**b**), and for Au-integrated PA in (**c**,**d**) obtained by varying h3, respectively. (**a**,**c**) under amorphous state and (**b**,**d**) under crystalline state.

Figure 5 shows the absorption spectra for Al-integrated and Au-integrated PAs as h3 increases from 30 nm to 100 nm. Figure 5a,b presents the absorption spectra of Al-integrated PA: the absorption peak is located around 5.7 μm under the amorphous state, while it is located around 7.2 μm under the crystalline state. Figure 5c,d presents the absorption spectra of Au-integrated PA: the absorption peak is located around 6.8 μm under the amorphous state, while it is located around 8.3 μm under the crystalline state. Clearly, the wavelength corresponding to the absorption peak is less relevant to the value of h3. Instead, it depends more on the electric permittivity of the utilized metal. As shown in Figure 5a–d, the absorption peak approaches unity for h_3_ values greater than 50 nm. Therefore, h3 = 70 nm is chosen to further study the absorption performance of PAs in the following simulations.

**Figure 6:**
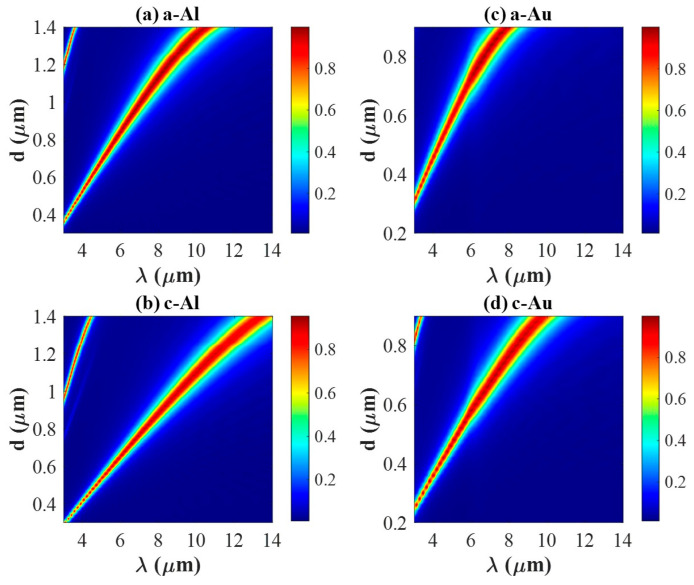
The absorption spectra for Al-integrated PA obtained by varying d from 0.3 μm to 1.4 μm in (**a**,**b**), for Au-integrated PA obtained by varying d from 0.2 μm to 0.9 μm in (**c**,**d**). (**a**,**c**) under amorphous state and (**b**,**d**) under crystalline state.

Figure 6 demonstrates the evolution of absorption spectra as d ranges from 0.3 to 1.4 μm for Al-integrated PA ((a) and (b)), and d varies from 0.2 to 0.9 μm for Au-integrated PAs ((c) and (d)), respectively, under two states. As shown in Figure 6a, no distinct absorption peak is observed for the Al-integrated PA when d is smaller than 0.36 μm. Similarly, for the Au-integrated PA (Figure 6c), a resonance emerges only when d exceeds approximately 0.3 μm. This absence of resonance at small d values may be due to the top antenna being too small to form a resonance in the range of 3–14 μm. The absorption peak reaches unity as long as it shows up, and it is extremely red-shifted as d scales up for both metals, which agrees with the theoretical prediction on the resonant properties of the dielectric resonator [37].

Moreover, Figure 6a demonstrates two peaks emerging as d is longer than 1.2 μm, and Figure 6b presents two peaks emerging as d is longer than 1 μm. Increasing the size of the top antenna results in a longer effective optical path length. Once d reaches a critical value, it results in the enhancement of a secondary resonance at a longer wavelength [37], one side peak emerges. To distinguish the difference between the side peak and main peak, we first analyzed the absorption spectrum for Al-integrated PA with d = 1.4 μm under both crystalline and amorphous states, which possess two peaks (Figure S1). The side peak is both lower in intensity and narrower in spectral width than the main resonance peaks shown in Figure 2. Secondly, we also investigated the electromagnetic distribution of the two peaks in Figure S1, where the main peak looks similar to those shown in Figure 3 and Figure 4. However, it demonstrates two more nodes for the side peak in the dielectric cavity (Figures S2 and S3), and a higher-order resonance mode is formed.

### 3.4. Crystallization Levels

Even though the resonance wavelength is sensitive to d and varies from 3 μm to 10 μm, which encompasses the majority of the infrared atmospheric window (3 μm to 14 μm), this geometric tuning approach is static and irreversible after fabrication. Therefore, active tunability must be achieved through the phase transition of Sb_2_S_3_, whose optical properties differ dramatically between the amorphous and crystalline states across the visible to infrared spectrum. The Sb_2_S_3_ layer can be transferred between amorphous and crystalline states via stimulation with either electrical or laser pulses. In addition, partially crystalline intermediate states often exist between the amorphous state and the crystalline state [46,47]. Therefore, the optical response of the PA can be gradually adjusted by applying different energies of pulses during the transfer process [48]. To describe the effective permittivity of Sb_2_S_3_ with various fractions of crystallization, we used the Lorentz–Lorenz effective medium theory [49]:

(1) $$\frac{\varepsilon_{eff}\left(\lambda \right)-1}{\varepsilon_{eff}\left(\lambda \right)+2}=m\;*\;\frac{\varepsilon_{c}\left(\lambda \right)-1}{\varepsilon_{c}\left(\lambda \right)+2}+(1-m)\;*\;\frac{\varepsilon_{a}(\lambda )-1}{\varepsilon_{a}(\lambda )+2}$$

where $\varepsilon_{a}(\lambda )$ and $\varepsilon_{c}(\lambda )$ are the corresponding permittivities of amorphous Sb_2_S_3_ and crystalline Sb_2_S_3_ as a function of the wavelength (λ), respectively. m is defined as the crystallization fraction of Sb_2_S_3_, ranging from 0 to 1, where 0 stands for the amorphous state and 100% stands for the crystalline state.

**Figure 7:**
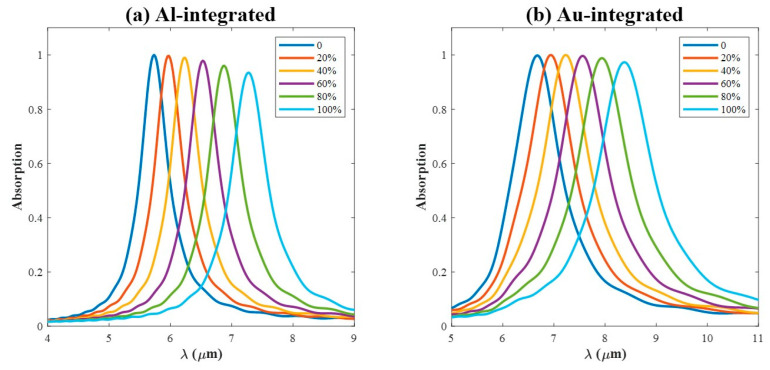
The absorption spectrum for different crystallization levels ranging from 0 to 100% for Al-integrated PA in (**a**) and for Au-integrated PA in (**b**). 0 stands for the amorphous state, and 100% stands for the crystalline state.

For a given crystallization fraction m, the effective refractive index $n_{eff}$ is determined as a function of wavelength through Equation (1). Figure 7 shows the evolution of the absorption spectrum, and the resonance peak is red-shifted as m increases, which is consistent with the results in Figure 2. For the Al-integrated PA (Figure 7a), the amplitude of the resonance peak gradually decreases with increasing m. In contrast, for the Au-integrated PA (Figure 7b), the amplitude initially exhibits a slight increase, followed by a gradual decrease as m rises. This may be due to the strongest absorption can only be excited when the LSPRs and the dielectric cavity resonance coincide with each other [41,42].

**Figure 8:**
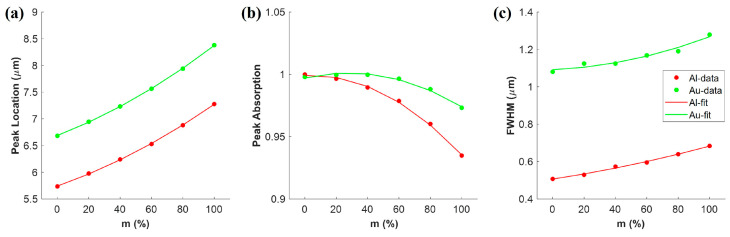
(**a**) Location, (**b**) absorption value, and (**c**) FWHM of peak vs. crystallization levels (m). Red color stands for Al-integrated PA, while green color stands for Au-integrated PA. Dots present for simulation data and lines present for fitted data.

We further numerically analyzed behaviors of the resonance wavelength, peak absorption, and full width at half maximum (FWHM) with respect to crystallization fraction m, shown in Figure 8a. The spectral data and corresponding fitting curves are color-coded: red represents results for the Al-integrated PA, while green denotes those for the Au-integrated PA. The resonance wavelength for each structure was accurately described by a second-order polynomial function of m. For Al-integrated PA, it is as follows:

(2) $$\lambda_{Al}=5.7406+1.0285m+0.50192m^{2}\;\;(\mu m)$$

This equation can be used to modulate any peak as a function of m between 5.73 μm and 7.28 μm. For Au-integrated PA, the fitted line is valid for wavelengths from 6.68 μm to 8.38 μm, and it is as follows:

(3) $$\lambda_{Au}=6.6877+1.1335m+0.55125m^{2}\;\;\;\;(\mu m).$$

Figure 8b reveals peak absorption decreasing as m augments for Al-integrated PA. The red fitted line is expressed as follows:

(4) $$A_{Al}=0.9991-0.0058m-0.0695m^{2}$$

The Au-integrated PA exhibits a non-monotonic trend in peak absorption, which initially experiences a slight increase before undergoing a gradual decrease as m rises. Its fitting line is as follows:

(5) $$A_{Au}=0.9970+0.0294m-0.0524m^{2}$$

Figure 8c demonstrates that the peak broadens as m increases. FWHM can also be fitted by a two-order polynomial function of m. For Al-integrated PA, it is as follows:

(6) $${FWHM}_{Al}=0.50623+0.12711m-0.049237m^{2}\;\;\;\;(\mu m).$$

And for Au-integrated PA, it is as follows:

(7) $${FWHM}_{Au}=1.0912+0.038261m-0.13813m^{2}\;\;(\mu m).$$

It is evident that the peaks broaden as m augments, which may be due to the increased damping of the plasmon resonance [50,51]. All in all, all the absorptions are larger than 93% as m changes from 0 to 1, which is comparable with the reported results [12,15,28] and much better than the results in [15]. Consequently, it is possible to control the resonance wavelength in a relatively wide range by controlling the crystallization level in the Sb_2_S_3_ layer with high absorption.

### 3.5. Incident Angle Dependent

**Figure 9:**
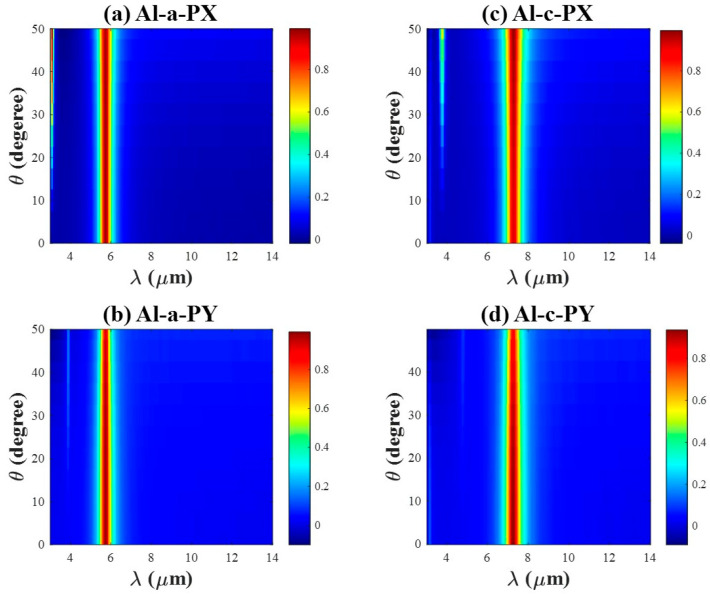
The absorption spectrum with the incident angle varying from 0 to 50 degrees for Al-integrated PA. (**a**,**c**) with TE incidence, and (**b**,**d**) with TM incidence. (**a**,**b**) under the aSb_2_S_3_ state, while (**c**,**d**) under the cSb_2_S_3_ state.

**Figure 10:**
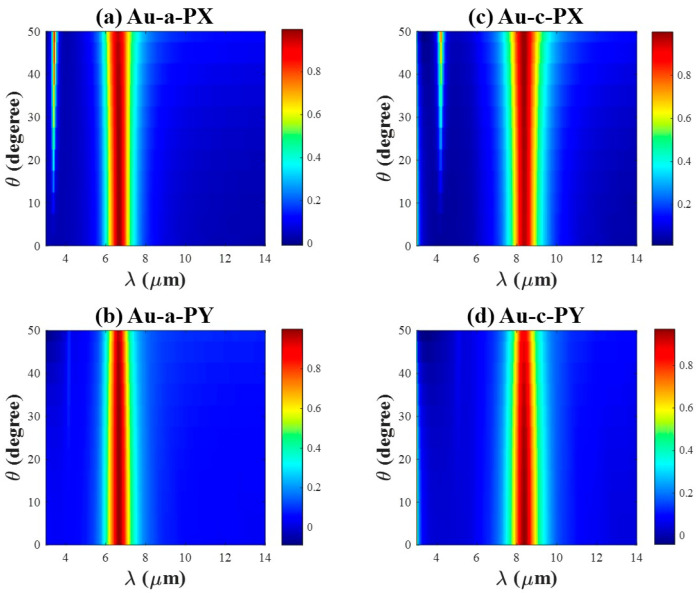
The absorption spectrum with the incident angle varying from 0 to 50 degrees for Au-integrated PA. (**a**,**c**) with TE incidence, and (**b**,**d**) with TM incidence. (**a**,**b**) under aSb_2_S_3_ state, while (**c**,**d**) under cSb_2_S_3_ state.

The above discussions take place with normal incident light, and the absorption responses as a function of the incident elevation angle are also analyzed. Figure 9 and Figure 10 demonstrate the simulated absorption spectrum for TE and TM for Al/Au-integrated PA in (a, b) under the Sb2S3 state, and in (c, d) under the cSb_2_S_3_ state. The resonance wavelength remains nearly constant with increasing angle, and the peak absorption amplitude shows only a modest reduction even at large angles of incidence. One side peak emerges for all cases as the incident elevation angle enlarges, whose amplitude is relatively large for TE incidence but small for TM incidence. Furthermore, the absorption spectrum broadens considerably with increasing angle for TE polarization. In contrast, it narrows under TM polarization.

Given that the angular dependence exhibits similar trends for both phase states, the following comparison focuses on the characteristics of the selected resonance peaks under the amorphous (aSb_2_S_3_) state. For Al-integrated PA, the absorption peak is 99.9% and FWHM is 507.02 nm for both TE and TM incidence at an incident angle of 0° due to the structure’s symmetry. At an incident angle of 50°, the FWHM broadens to 608.04 nm for TE polarization, whereas it narrows to 386.94 nm for TM polarization. Under the same oblique incidence (50°), the peak absorption decreases to 98.8% for TE polarization and to 93.3% for TM polarization. For Au-integrated PA, FWHM is 1080.2 nm for both TE and TM at normal incidence. Under TE polarization at 50°, the FWHM broadens to 1326.6 nm, and the peak absorption decreases to 95.4%. Notably, this reduction in absorption remains relatively modest compared to values reported in other studies [12,15]. In contrast, under TM polarization, the FWHM narrows to 884.4 nm and the peak absorption reduces to 99.3% at 50°.

In addition, we demonstrate the electromagnetic distribution corresponding to the main and side peaks at an incident elevation angle of 40° for Al/Au-integrated PA in Supporting. The electromagnetic field profiles of the main peaks closely resemble those shown in Figure 3 and Figure 4. In contrast, two nodes display in the electromagnetic field pattern of the side peaks, corresponding to the second-order resonance mode. The appearance of the side peak may be attributed to the enlarged optical path as theta increases, which facilitates the excitation of a higher-order resonance mode (see Supporting Information). The electromagnetic energy is mainly confined within the dielectric cavity and is efficiently absorbed even at large incidence angles [52].

In brief, the proposed metasurface is polarization-insensitive over a wide range of incident angles across the infrared region, which is ideal for several practical applications such as thermometers, bolometers, and so on [11]. Additionally, this design concept can also be applied to other phase change materials, e.g., Sb-Te alloy [53].

## 4. Conclusions

In summary, we numerically demonstrated a tunable perfect narrow-band MIM-structured metasurface perfect absorber in the infrared region. The absorber consists of a periodic array of Al or Au nanodisks positioned above a continuous Sb_2_S_3_ layer and a bottom Al or Au film. A notable resonance shift of up to 1.5 μm was achieved between the amorphous and crystalline phases of Sb_2_S_3_. Near-field distribution of both electric and magnetic fields has been displayed to show that the perfect absorption is caused by the simultaneous electric dipole and magnetic resonance, attributing to the coupling effect of LSPR of metasurface and cavity resonance. Furthermore, the absorption resonance wavelength is highly sensitive to the side length of the metal antenna, but insensitive to the thickness of Sb_2_S_3_. Moreover, the absorption amplitude maintains high efficiency across a wide range of incident angles. This design provides an alternative solution for tunable perfect absorbers and will have potential applications in reconfigurable photonic devices for energy harvesting and infrared sensing.

## Data Availability

The original contributions presented in this study are included in the article/Supplementary Material. Further inquiries can be directed to the corresponding authors.

## Supplementary Materials

The following supporting information can be downloaded at: https://www.mdpi.com/article/10.3390/ma18235305/s1, Figure S1: Absorption spectrum of Al-integrated PA with p = 1.5 µm and d = 1.4 µm. Blue line: amorphous Sb_2_S_3_; Orange line: crystalline Sb2S3; Figure S2: (a–c) and (d–f) E(xy), E(xz) and H(xz) corresponding to peak A and C in Figure S1, respectively; Figure S3: (a–c) and (d–f) E(xy), E(xz) and H(xz) corresponding to peak B and D in Figure S1, respectively; Figure S4: Absorption spectrum at incident angle θ = 40° under aSb_2_S_3_ state and x-polarization. Blue line: Al-integrated PA; Red line: Au-integrated PA; Figure S5: (a–c) and (d–f) E(xy), E(xz) and H(xz) corresponding to peak G and E in Figure S4, respectively; Figure S6: (a–c) and (d–f) E(xy), E(xz) and H(xz) corresponding to peak H and F in Figure S4, respectively; Figure S7: Absorption spectrum at incident angle θ = 40° under aSb_2_S_3_ state and y-polarization. Blue line: Al-integrated PA; Red line: Au-integrated PA; Figure S8: (a–c) and (d–f) E(xy) E(yz) and H(yz) of peak K and I in Figure S7, respectively; Figure S9: (a–c) and (d–f) E(xy) E(yz) and H(yz) of peak J and L in Figure S7, respectively. Figure S10. E(yz) of peak J in yz plane at x = 0.4 (a), 0.3 (b), −0.3 (c) and −0.4 (d) µm; Figure S11. H(yz) of peak J in yz plane at x = 0.4 (a), 0.3 (b), −0.3 (c) and −0.4 (d) µm; Figure S12. E(yz) of peak I in yz plane at x = 0.4 (a), 0.3 (b), −0.3 (c) and −0.4 (d) µm; Figure S13. H(yz) of peak I in yz plane at x = 0.4 (a), 0.3 (b), −0.3 (c) and −0.4 (d) µm.
