# Supplementary material for: Improvement of a Switchable Wide-Incident-Angle Perfect Absorber Incorporating Sb2S3
Source: Materials (Basel). 2025 Nov 25;18(23):5305. doi: 10.3390/ma18235305 (PMC12692817; doi:10.3390/ma18235305)
Supplement: Supplementary file 1 [file materials-18-05305-s001.zip › materials-3920173-supplementary.pdf]

Supporting Information

Improvement of a Switchable Wide-Incident-Angle Perfect Absorber Incorporating Sb_2S_3

Yaolan Tian [1,†], Guoxu Zhang [1,†], Yan Li[1], Mei Shen[2], Yufeng Xiong[1], Ting Li[1], Yunzheng Wang[1,3,4]*, Xian Zhao[1]* and ChangBao Ma[1]

[1]Center for Optics Research and Engineering, Shandong Provincial Key Laboratory of Laser Technology and Application, Key Laboratory of Laser & Infrared System, Ministry of Education, Shandong University, Qingdao, 266237, China;
[2]College of Integrated Circuits, Shenzhen Polytechnic University, Shenzhen 518052, China
[3]Suzhou Research Institute of Shandong University, Room1107, Building B of NUSP, NO.388 Ruoshui Road, SIP, Suzhou, Jiangsu, 215123, China
[4]Shenzhen Research Institute of Shandong University, A301 Virtual University Park in South District of Shenzhen, Shenzhen, 518055, China

*Correspondence: YZW: yunzheng_wang@sdu.edu.cn, XZ: zhaoxian@sdu.edu.cn
† These authors contributed equally to this work.

1. Absorption spectrum for Al-integrated PA with p=1.5 μm and d=1.4 μm.

Figure S1 illustrates the absorption spectrum for Al-integrated PA with p = 1.5 μm and d = 1.4μm under both amorphous state (blue line) and crystalline state (orange line). Two peaks arise for each spectrum. Both the main resonance peak (C) and the side peak (A) are red shifted as Sb_2S_3 transferred from amorphous state to crystalline state. Besides, the main peaks are broader and higher than the side peaks. Under aSb_2S_3 state, amplitude of peak C is 97.7%, locating at 10.48μm, while it is 84.7% for peak A, centering at 3.60 μm. FWHM of peak C is 1.45 μm, in the contrast, it is 0.12 μm for peak A. Under cSb_2S_3 state, the main peak D is as large as 86.5%, centering at 13.4 μm, while peak B is as high as 75.1% seats at 4.45 μm. In addition, FWHM of peak D is 1.93μm, but it is 0.181 μm for the peak B.

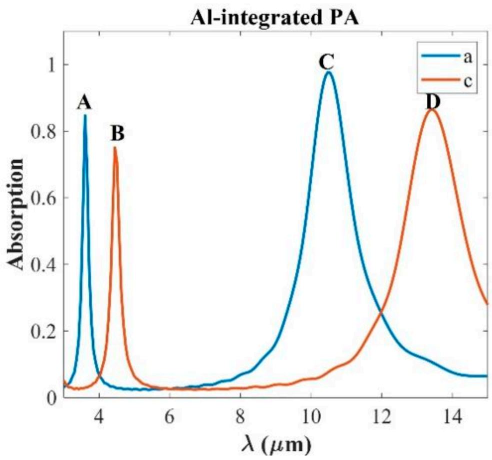

Figure S1. Absorption spectrum of Al-integrated PA with p = 1.5 μm and d = 1.4 μm. Blue line: amorphous Sb_2S_3; Orange line: crystalline Sb_2S_3.

The near field electromagnetic fields of four peaks (A, B, C and D) in **Figure S1** are investigated. **Figure 2 (a)-(c)** show the electromagnetic field distribution of peak A, and **(d)-(f)** present that of peak C. More specifically, **(a)(d)** shows electric field (E(xy)) in the x-y plane on the surface of Ge layer, **(b)(e)** and **(c)(f)** show the electric field distribution (E(xz)) and the magnetic distribution (H(xz)) respectively in the x-z plane when y=0, i.e. the x-z plane goes through the middle line of a unit cell.

The electromagnetic field distribution mode of peak C (**Figure S2(d)-(f)**) are the same as those in **Figure 3** and **4** in our main paper. However, the strength of electromagnetic field in **Figure S2(d)-(f)**) is a bit weaker than that in **Figure 3,** which means the confined energy is less than that in **Figure 3**. Consequently, the amplitude of Peak C in **Figure S1** is smaller than that of peak A in **Figure 2(a)**. **Figure S2(a)-(c)** show three nodes emerge in the electromagnetic field distribution, due to the dielectric cavity resonance, thus it is 3rd mode[1]. The 1st mode requires a relatively short optical path, and it can show up when d is small[2-3]. As d enlarge, the optical path in the cavity enlarges, and thus higher cavity mode can be activated, thus high-order mode emerges. **Figure S2 (a) (b)** show the electric field is mainly distributed as electric dipole, and confined around the edge of the top antenna along y axis due to coupling effect of adjacent antennas and LSPRs[4]. Meanwhile, under this resonance mode, three loops of displacement current (**Figure S2 (c)**) are formed, whose circle direction of adjacent ones are opposite. Those loops dominantly contribute to three nodes in electric field, and a 3rd mode distribution of H(xz)[1-2, 5-6]. It is can be concluded that the coupling effect of the electric dipole and cavity resonance contribute to the absorption peak.

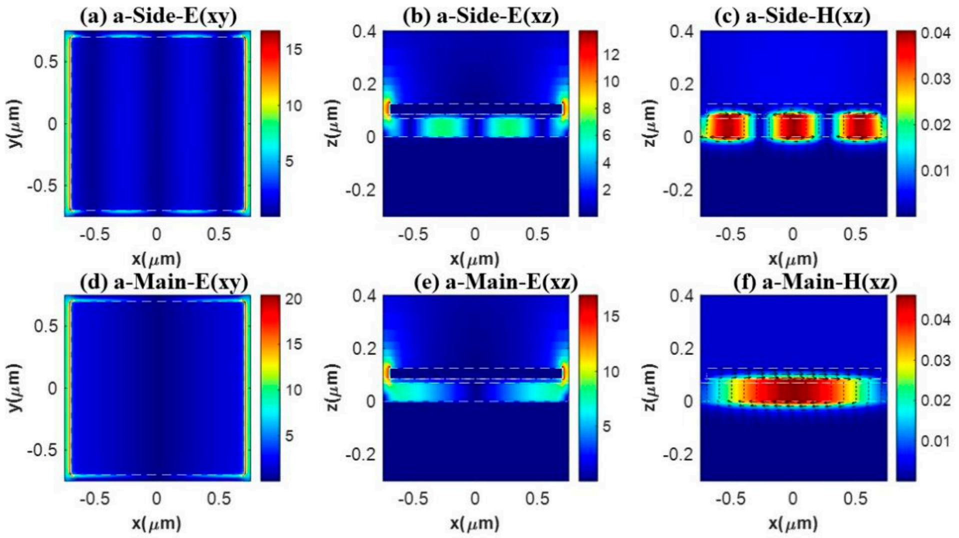

Figure S2. (a)-(c) and **(d)-(f)** E(xy), E(xz) and H(xz) corresponding to peak A and C in **Figure S1**, respectively.

Figure S3 presents the electromagnetic field patterns corresponding to peak B and D in **Figure S1**. Those patterns are quite similar with the ones in **Figure S2**. However, the electromagnetic strength in **Figure S3** is weaker than that in **Figure S2** (Strength: peak A > peak B, peak C>peak D), which leads to a lower absorption.

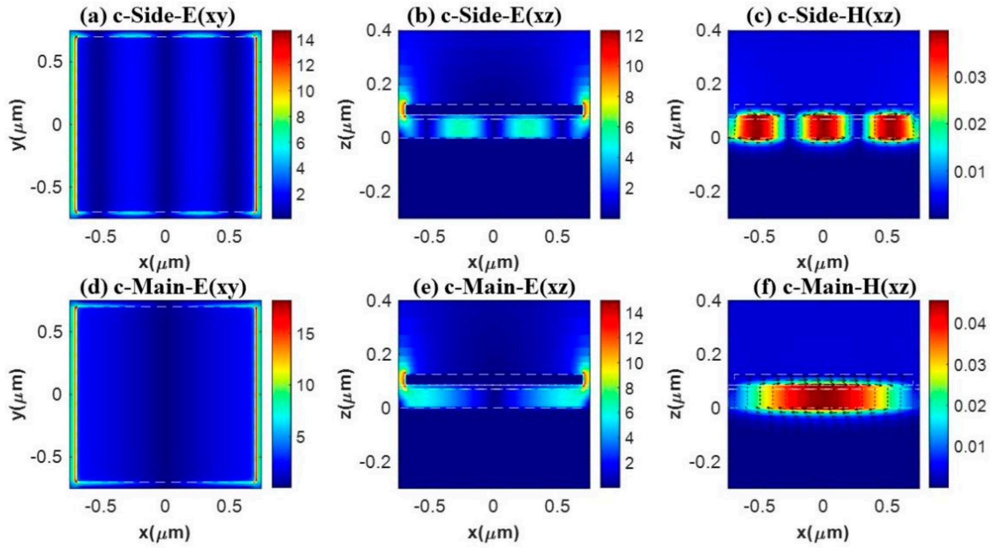

Figure S3. (a)-(c) and **(d)-(f)** E(xy), E(xz) and H(xz) corresponding to peak B and D in **Figure S1**, respectively.

2. Absorption profile for Al/Au-integrated PA with d=0.8 μm at incident angle 40°.

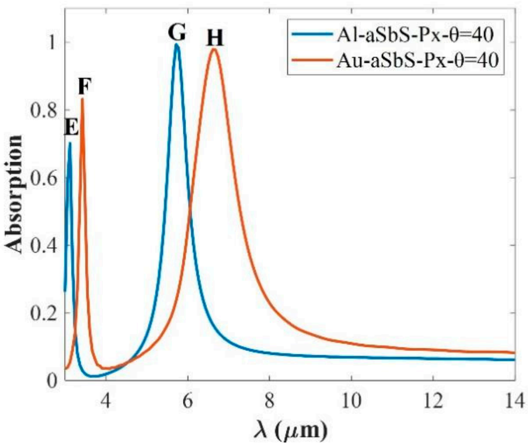

Figure S4. Absorption spectrum at incident angle θ = 40° under aSb_2S_3 state and x-polarization. Blue line: Al-integrated PA; Red line: Au-integrated PA.

Figure S4 demonstrates the absorption spectrum when incident angle θ=40° under aSb_2S_3 state. The blue line stands for Al-integrated PA, while the orange one stands for Au-integrated PA, respectively. For Al-integrated PA, the main peak (G) is broadener and higher than the side peak (E). Peak G locates at 5.71μm of a value 99.3% and FWHM 543 nm. Its corresponding electromagnetic field are shown in **Figure S5 (a)-(c),** evidently, which are similarly with those generated with normal incident (**Figure 3,4** and **Figure S2,S3 (e)-(f)**). Peak E locates at 3.12 μm with an amplitude of 70.1% and FWHM 121 nm. **Figure S5 (d)-(f)** illustrates the electromagnetic field distribution of peak E, and two nodes present in E(xy) and E(xz), it is second-order mode. According, H(xz) is also second-order mode. As θ enlarged, the optical path for the light reflected in the cavity is lengthened, therefore, high-order resonance mode can be formed[3, 7].

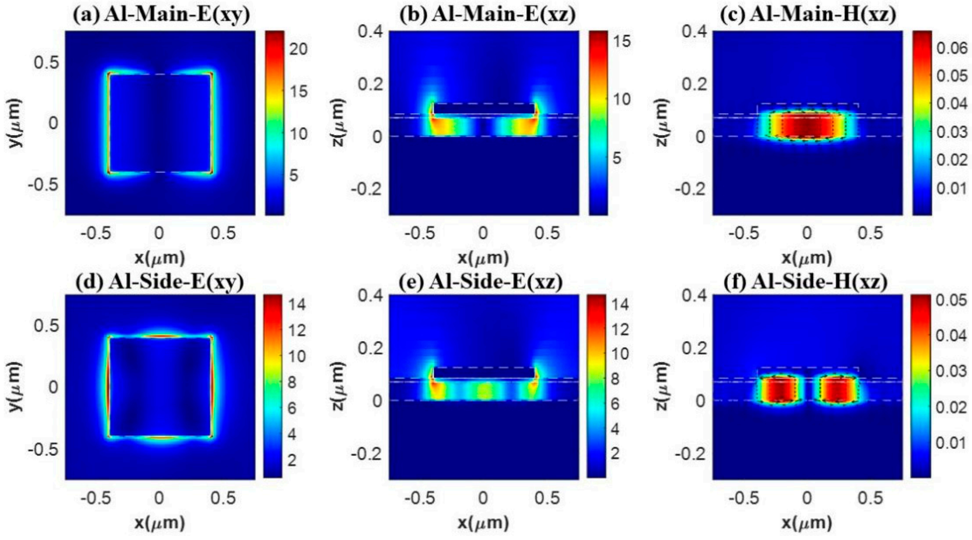

Figure S5. (a)-(c) and **(d)-(f)** E(xy), E(xz) and H(xz) corresponding to peak G and E in **Figure S4**, respectively.

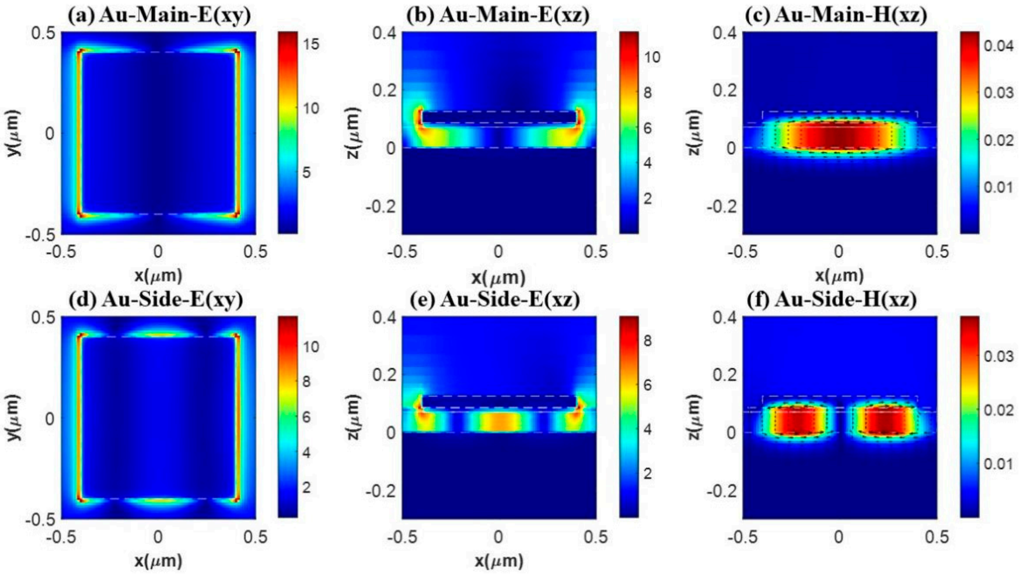

Figure S6. (a)-(c) and **(d)-(f)** E(xy), E(xz) and H(xz) corresponding to peak H and F in **Figure S4**, respectively.

For Au-integrated PA, peak H locates at 6.62 μm of a value 97.9%, and of FWHM 1206 nm. Its E(xy), E(xz) and H(xz) are illustrated in **Figure S6 (a)-(c).** Peak F seats at 3.42 um, of a value 83.2% and of FWHM 121 nm. Accordingly, its E(xy), E(xz) and H(xz) are illustrated in **Figure S6 (d)-(f),** which are similarly as those in **Figure S5**.

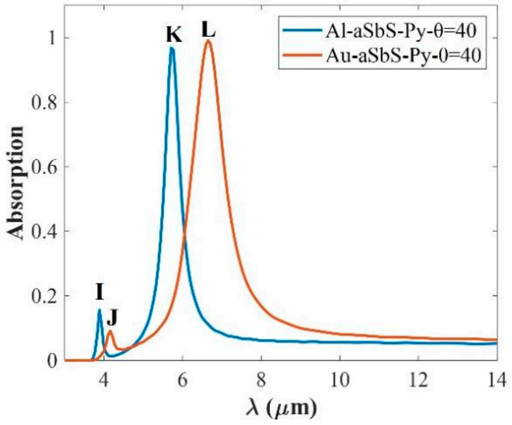

Figure S7. Absorption spectrum at incident angle θ = 40° under aSb_2S_3 state and y-polarization. Blue line: Al-integrated PA; Red line: Au-integrated PA.

Figure S7 demonstrates the absorption spectrum when incident angle θ=40° with y-polarization under aSb_2S_3 state. The blue one stands for Al-integrated PA, while the orange one stands for Au-integrated PA, respectively. In this case, the side peaks are highly reduced than the main peaks. For Al-integrated PA, the side peak I locates at 3.88 μm of a value 15.6%, while the main peak K locates at 5.71 μm with a value of 96.9%. For Au-integrated PA, the side peak J locates at 4.16 μm, and is of a value 9.1%, while the main peak L locates at 6.65 μm with a value of 99.1%.

The corresponding electromagnetic fields of peak I,J,K,L are investigated on the surface of Ge layer (E(xy)), on the plane yz going through middle of a unit cell (E(yz) and H(yz)). **Figure S8,S9 (a)-(c)** show E(xy), E(yz) and H(yz) of peak K,L, which is pretty the same as those of the main peaks demonstrated in the above. **Figure S8,S9 (d)-(f)** presents E(xy), E(yz) and H(yz) of peak I,J. It is evident that E(xy) in **(d)** is not an electric dipole anymore, instead it is an asymmetry electric quadrupole due to TE-incidence. E(yz) in **(e)** is mainly confined in the Sb_2S_3 layer, as well as reflected in the above air. Besides, the strength of E(yz) is pretty smaller than that in **(b)**. Consequently, no magnetic dipole displays in H(yz) in **Figure S8,S9 (f)**. The unique behavior of E(yz) and H(yz) in **Figure S8,Figure S9** is mostly probable attribute to the selection of x at 0, which approaches the electric node.

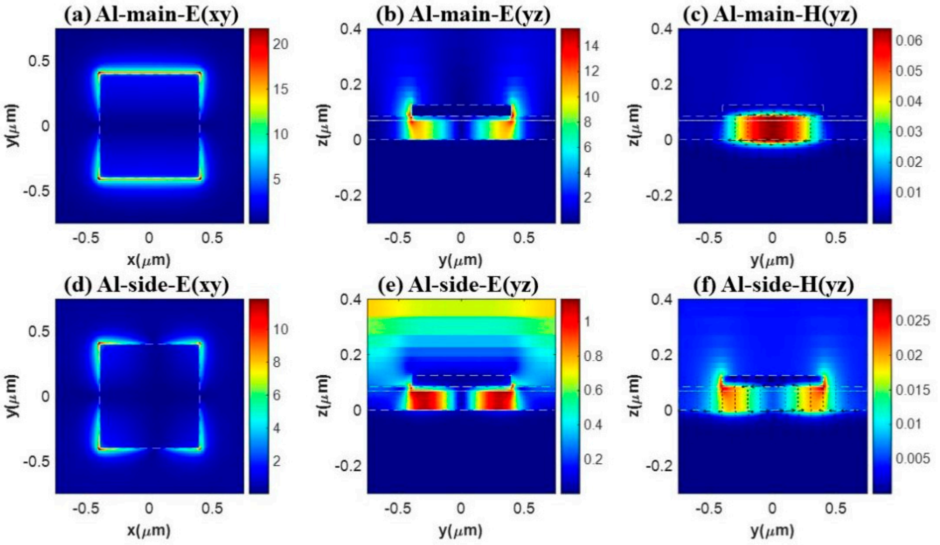

Figure S8. (a)-(c) and **(d)-(f)** E(xy) E(yz) and H(yz) of peak K and I in **Figure S7**, respectively.

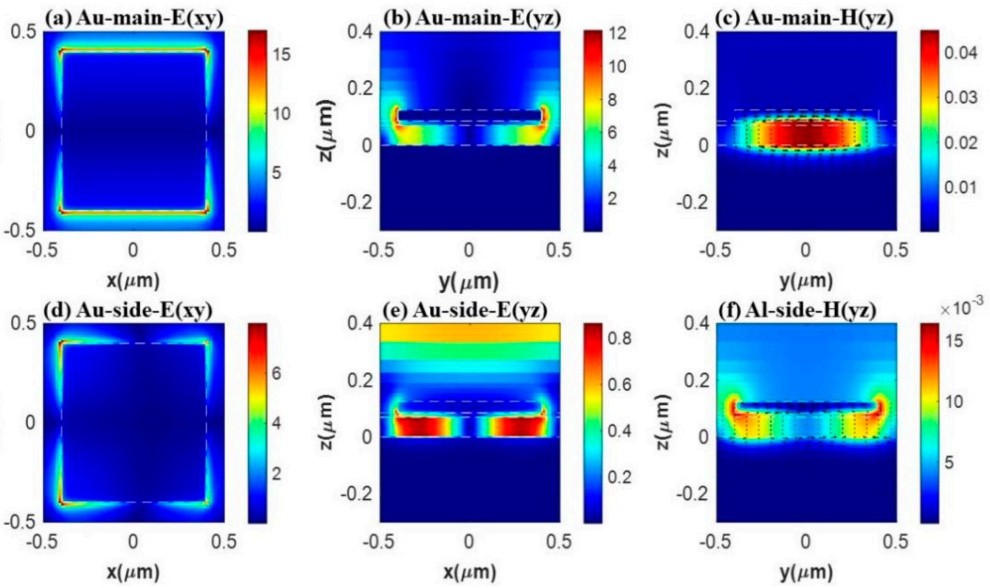

Figure S9. (a)-(c) and **(d)-(f)** E(xy) E(yz) and H(yz) of peak J and L in **Figure S7**,

respectively.

To further figure out the reason of forming a small side peak, electromagnetic field distributions in yz plane at x=0.4,0.3,-0.3,-0.4 μm are investigated. **Figure S10,S11** demonstrate the electromagnetic field distributions of peak J, while **Figure S12,S13** display the electromagnetic distributions of peak I. **Figure S10,S12** show that E(yz) looks different as in **Figure S8(e),S9(e),** but similarly as that in **Figure S8(b),S9(b)**. The node does not locate in the center, and the strength of E(yz) is dependent on x.

Figure S11,S13 indicate that the magnetic field distributions at x=-0.4,-0.3 μm and x=0.4,0.3 μm are slightly different. **(a)(b)** show H(yz) locates around the interface of top antenna and Ge layer, while **(c)(d)** show H(yz) locates inside dielectric. The small amplitude of peak I,J may due to the electric quadrupole and no magnetic resonance.

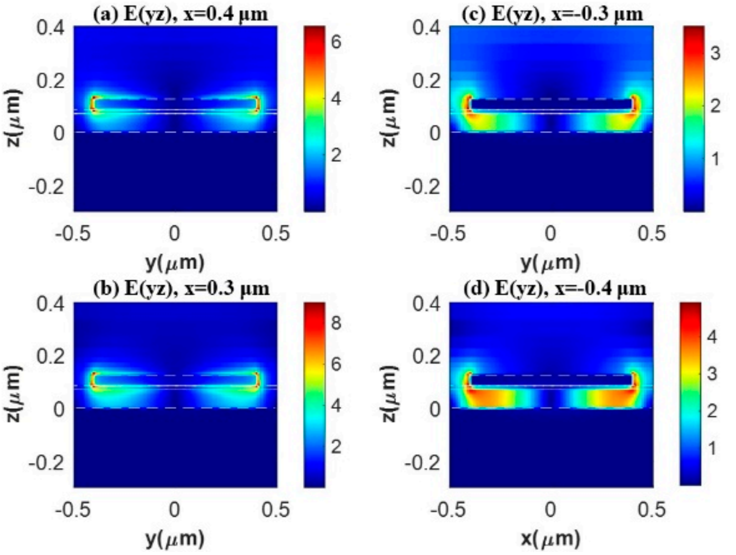

Figure S10. E(yz) of peak J in yz plane at x = 0.4**(a),** 0.3**(b),** -0.3**(c)** and -0.4**(d)** μm.

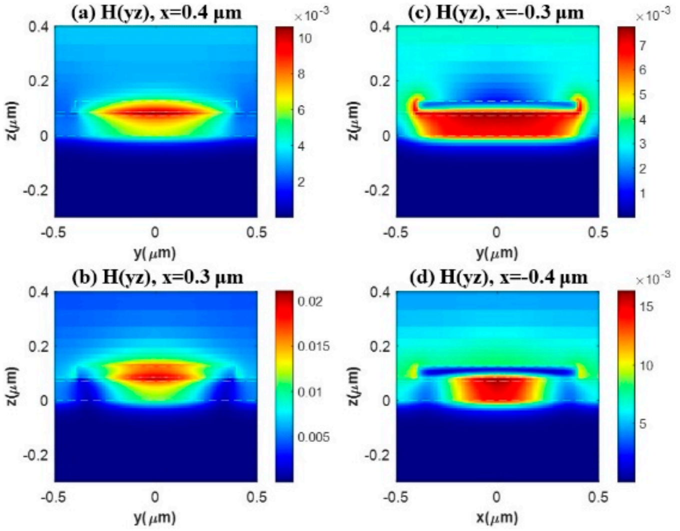

Figure S11. H(yz) of peak J in yz plane at x = 0.4**(a),** 0.3**(b),** -0.3**(c)** and -0.4**(d)** μm.

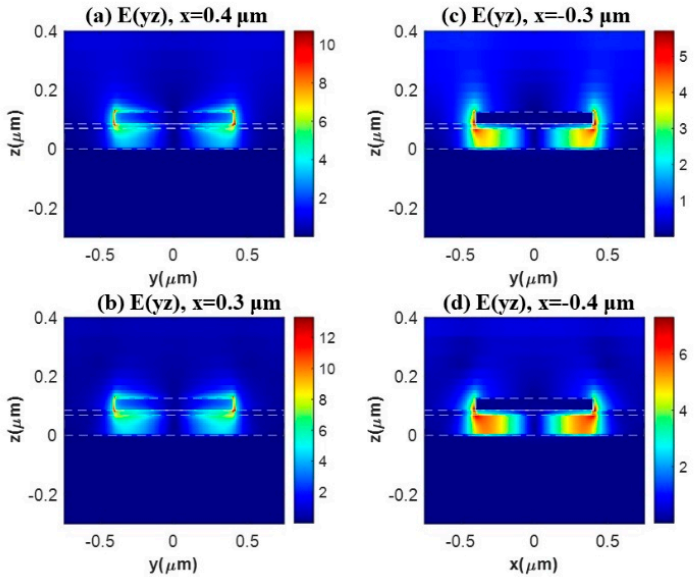

Figure S12. E(yz) of peak I in yz plane at x = 0.4**(a),** 0.3**(b),** -0.3**(c)** and -0.4**(d)** μm.

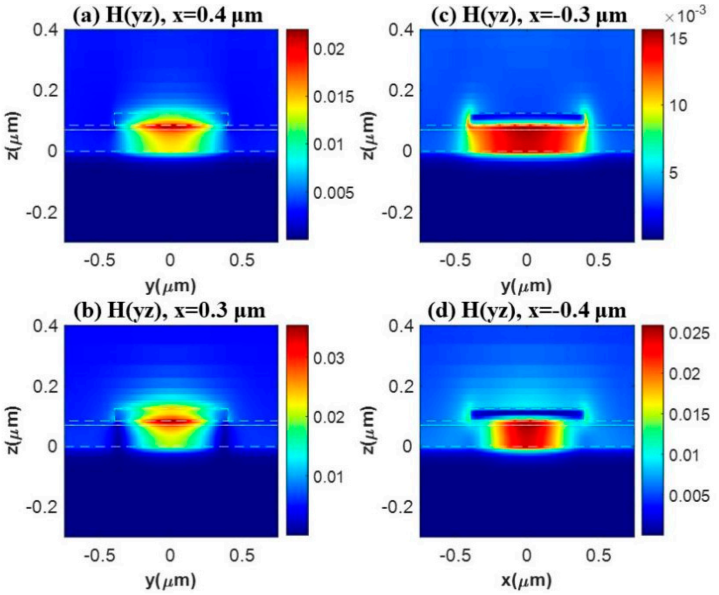

Figure S13. H(yz) of peak I in yz plane at x = 0.4**(a),** 0.3**(b),** -0.3**(c)** and -0.4**(d)** μm.

References

[1] Y. Zhu, T. Lan, P. Liu, J. Yang, Broadband near-infrared TiO2 dielectric metamaterial absorbers, *Applied Optics* **2019**,58, 7134-7138.

[2] T. Cao, L. Zhang, R. E. Simpson, M. J. Cryan, Mid-infrared tunable polarization-independent perfect absorber using a phase-change metamaterial, *Journal of the Optical Society of America B* **2013**,30, 1580-1585.

[3] R. Ameling, H. Giessen, Cavity Plasmonics: Large Normal Mode Splitting of Electric and Magnetic Particle Plasmons Induced by a Photonic Microcavity, *Nano Letters* **2010**,10, 4394-4398.

[4] T. Cao, X. Zhang, W. Dong, L. Lu, X. Zhou, X. Zhuang, J. Deng, X. Cheng, G. Li, R. E.

Simpson, Tuneable Thermal Emission Using Chalcogenide Metasurface, *Advanced Optical Materials* **2018**,6, 1800169.
[5] M. Zhu, S. Abdollahramezani, C. Li, T. Fan, H. Harutyunyan, A. Adibi, Dynamically tunable second-harmonic generation using hybrid nanostructures incorporating phase-change chalcogenides, *Nanophotonics* **2022**,11, 2727-2735.
[6] S. Zhang, K. Zhou, Q. Cheng, L. Lu, B. Li, J. Song, Z. Luo, Tunable narrowband shortwave-infrared absorber made of a nanodisk-based metasurface and a phase-change material Ge2Sb2Te5 layer, *Applied Optics* **2020**,59, 6309-6314.
[7] Z. Liu, G. Liu, G. Fu, X. Liu, Y. Wang, Multi-band light perfect absorption by a metal layer-coupled dielectric metamaterial, *Optics Express* **2016**,24, 5020-5025.
